# Machine learning-based ultrasound radiomics for prediction of 6-month local thermal ablation response in benign thyroid nodules: a multicenter study

**DOI:** 10.3389/fendo.2026.1831990

**Published:** 2026-08-18

**Authors:** Xiaowan Bo, Zhuoran Wang, Chongke Zhao, Chaonan Li, Songyuan Yu, Jing Guo, Huihui Chai, Chengzhong Peng, Jiangang Chen, Jun Luo, Liping Sun, Huixiong Xu

**Affiliations:** 1 Department of Medical Ultrasound, Center of Minimally Invasive Treatment for Tumor, Shanghai Tenth People’s Hospital, Ultrasound Research and Education Institute, Clinical Research Center for Interventional Medicine, School of Medicine, Tongji University, Shanghai, China; 2 Shanghai Engineering Research Center of Ultrasound Diagnosis and Treatment, Shanghai, China; 3 Shanghai Key Laboratory of Multidimensional Information Processing, East China Normal University, Shanghai, China; 4 Department of Ultrasound, Zhongshan Hospital, Institute of Ultrasound in Medicine and Engineering, Fudan University, Shanghai, China; 5 Department of Ultrasound, Sichuan Provincial People’s Hospital, University of Electronic Science and Technology of China, Chengdu, China

**Keywords:** benign thyroid nodules, machine learning, radiomics, thermal ablation, ultrasound

## Abstract

**Objective:**

This study aimed to develop a machine learning (ML)-based ultrasound (US) radiomics model for prediction of 6-month local treatment response (LTR) ofthermal ablation (TA) for benign thyroid nodules (BTNs).

**Methods:**

Between January 2018 and July 2021, a total of 388 patients who underwent US-guided TA in three centers were included. US radiomics features were extracted from preoperative grayscale US images and data dimensionality reduced using principal component analysis and least absolute shrinkage and selection operator. Then, support vector machine (SVM), logistic regression, a decision tree, K-nearest neighbors, and random forest were applied to selected key US radiomics features for distinguishing a volume reduction ratio (VRR) ≥50% or <50%. Factors affecting 6-month LTR were assessed using multivariate logistic regression to construct a clinical model. Receiver operating characteristic curves were plotted to compare the predictive performance between radiomics-based ML and clinical models.

**Results:**

At 6 months post-ablation, patients with VRR ≥ 50% were 75.8% (292/372) in the training and internal test cohorts and 59.4% (19/32) in the external test cohort. Finally, 10 US radiomics features were selected for analysis. Solidity was the only independent clinical predictor associated with VRR<50%. Among the five algorithms, the SVM model achieved the optimal predictive efficacy, with an area under the curve (AUC) = 0.81 in the internal test set. The AUC of the SVM-based US radiomics model was significantly higher than that of the clinical model in both internal (0.81 vs. 0.63, P< 0.05) and external test cohorts (0.77 vs. 0.54, P< 0.05).

**Conclusion:**

The SVM-based US radiomicsmodel yielded a satisfactory performance for predicting the 6-month LTR, outperforming the clinical model, and facilitating decision-making in favor of TA.

## 1 Introduction

Thyroid nodules (TNs) affect 1%–5% of the global population; 85%–93% of which are benign (1, 2). Benign thyroid nodules (BTNs) can simply be followed up without requiring any treatment. Nodules growing over time can cause symptoms of compression, aesthetic concerns, and neck discomfort and should be treated surgically. Previously, thyroidectomy was the only available option to address mass effects in such cases. However, many patients are concerned about permanent neck scars and long-term levothyroxine use after surgery. Additionally, some patients are concerned about the risk of related complications, such as hypoparathyroidism and hoarseness. Therefore, minimally invasive non-surgery techniques for BTN treatment are gaining increasing attention both from patients and clinicians.

In recent years, ultrasound (US)-guided thermal ablation (TA) methods such as radiofrequency ablation (RFA), microwave ablation (MWA), and laser ablation, have been demonstrated to be a reliable alternative to surgery because of its ability to induce a significant volume reduction in BTNs (3–8). Accordingly, several guidelines have endorsed TA’s efficacy and safety for BTN treatment (9–14). However, not all nodules are suitable for TA. A volume reduction ratio (VRR)< 50% and nodular recurrence are the main obstacles for its application, particularly because a low VRR can lead nodular regrowth (15, 16). Thus, predicting the local treatment response to TA is necessary to identify the best candidates. However, to date, there is no reliable method to predict its postoperative outcomes. Recently, machine learning (ML)-based US radiomics methods have shown significant potential in the identification of malignant TNs, prediction of cervical lymph node metastasis of thyroid cancer, and efficacy of neoadjuvant chemotherapy (17–21). Thus, ML-based radiomics has potential as predictive a tool.

Therefore, we hypothesized that an ML-based US radiomics model could predict the postoperative local treatment response (LTR) of TA for BTNs. This study aimed to establish a ML-based US radiomics model and evaluate its value in predicting the 6-month LTR of TA in BTNs.

## 2 Materials and methods

### 2.1 Patients

All patients included in this study attended three hospitals between January 31, 2018, and July 15, 2021. This retrospective study was approved by the local institutional review board (approval number: SHSY-IEC-4.1/21-366/01), which waived the need for informed consent. All patients signed a written informed consent form before TA. Inclusion criteria were: (1) BTNs confirmed by fine needle aspiration biopsy (FNAB) and a Bethesda II classification; (2) underwent RFA or MWA treatment; (3) neck compression symptoms, cosmetic problems, or discomfort symptoms (such as throat discomfort, foreign body sensation, neck pain, and cough); and (4) ≥ 6-month follow-up. Exclusion criteria were: (1) age<18 years; (2) no complete clinical data; (3); nodules with multiple ablations; and (4) poor quality of US images.

### 2.2 Pre-ablation assessment

All patients underwent grayscale US, color Doppler flow imaging (CDFI), laboratory examinations, FNAB, and cervical thyroid examinations before TA. The US equipment was a GE LogiQ E9 (GE Medical Systems, Milwaukee, Wisconsin, USA), equipped with an ML6–15 high-frequency probe (6–15 MHz), and Philips EPIQ7 (Philips Medical, Basso District, Washington, USA), equipped with an eL18–4 linear array transducer (4–18 MHz). For each examination, the largest transverse and longitudinal section static images of nodules were saved.

TN echogenicity included hyper-, iso-, hypo-, and mixed echogenicity. Nodules close to vital organs were defined as nodules<3 mm from the trachea, esophagus, carotid artery, and danger triangle. The internal structure was used to divide them into solid, microcystic, or cystic nodules (22). Nodule vascularity was structured into four categories: (1) peripheral intense vascularity; (2) peri- and intranodular intense vascularity; (3) peripheral weak vascularity and only detectable around the nodule; and (4) no vascularity. Nodule volume was calculated as volume=length×width×height×0.524 (23).

Laboratory examinations included routine blood tests, prothrombin time and activated partial thromboplastin time, thyrotropin, total triiodothyronine, total thyroxine, free triiodothyronine, free thyroxine, and calcitonin.

### 2.3 Thermal ablation

RF generators (480 KHz, STARmed, Seoul, Korea) and modified straight internally cooled electrodes (18G) with active tip lengths of 7 or 10 mm were used for RFA. The generator had a 35 W output. An ECO-100 microwave multi-function therapeutic instrument (Changcheng Microwave System Engineering Co. Ltd, Nanjing, China) and disposable microwave antenna (17G) with an active tip length of 3 mm were used for MWA. The output power setting was 30 or 35 W, the output frequency 2,450 MHz, and an internally cooled microwave antenna with normal saline was used for cold fluid circulation.

If the nodule was adjacent to vital organs, saline was injected between the nodule and vital structures to protect these structures during ablation. A contrast-enhanced US was performed immediately to assess the LTR to ablation. The US contrast agent used was Sonovue (Bracco, Milan, Italy). A complete response shows no enhancement of the ablation zone, whereas a nodule with residual area manifests as visible enhancement areas in the ablation zone. In such cases, additional ablation was performed on the residual area.

### 2.4 Follow-up

After ablation, patients were asked to follow-up at 1, 3, 6, and 12 months and additionally at 6-month intervals thereafter. The nodule volume, symptoms, cosmetic score, and thyroid function tests were evaluated and recorded during the follow-up period. Nodule volume was measured using the US. VRR was calculated as follows: VRR (%)=(initial volume-final volume)/initial volume×100. Sufficient ablation was considered for VRR ≥ 50% (23).

### 2.5 ML-based US radiomics model

#### 2.5.1 Region of interest segmentation

For each nodule, two independent radiologists (with ≥5 years’ experience in US examinations) used 3D Slicer software (v4.11) to delineate the region of interest (ROI) around the nodule outline on the largest transverse or longitudinal section of a grayscale US image. If the two radiologists had a different result, the final result would be decided together after discussion. For lesions with indistinct margins or acoustic halos, a ROI was drawn along the outermost edge of the acoustic halo. Nodules with unrecognizable borderlines failing consistent delineation by two radiologists were excluded.

To evaluate the consistency of ROI segmentation, we used intra- and inter-group consistency testing for analysis. For intra-group consistency, the image ROI was divided into two groups, and the feature value of the ROI region of the two groups compared with intra-class correlation coefficients (ICCs) for consistency. For inter-group consistency, ICCs were used to assess the repeatability of radiomic features in ROI labeling results from both radiologists. Features with ICC ≥ 0.8 can be considered reliable.

#### 2.5.2 Feature extraction

Before feature extraction, image preprocessing was conducted. First, each image underwent binarization by transforming it into a binary representation based on the ROI boundary and accurately obtaining the corresponding lesion mask. Subsequently, wavelet filtering, Laplacian Gaussian filtering, and edge enhancement filtering techniques were used for further image processing. This involved calculating both gradient and pixel intensity values to derive radiomics features specific to shadow groups. Finally, all radiomics features were standardized using Z-score normalization.

Features were extracted from 405 qualified grayscale US images using PyRadiomics (https://pyradiomics.readthedocs.io/en/latest/index.html). Extracted features included first-order, shape, grayscale co-occurrence matrix, grayscale size region matrix, grayscale run-length matrix, adjacent grayscale difference matrix, and grayscale dependence matrix features.

#### 2.5.3 Dimensionality reduction and normalization

Regression fitting was performed on training data and the most valuable features filtered out. Dimensionality reduction reduces data redundancy by decreasing the number of features and acquiring irrelevant primary variables. Principal component analysis (PCA) and least absolute shrinkage selection operator (Lasso) were used to reduce the dimensionality of high-dimensional data. The PCA algorithm considers all high-throughput features and reduces feature dimensions according to the selected coefficients before the Lasso algorithm extracts features with high correlation to those of the lesion region among the dimensionality reduction features. Normalization enables different features to be comparable.

#### 2.5.4 Model training and validation

**Figure 1:**
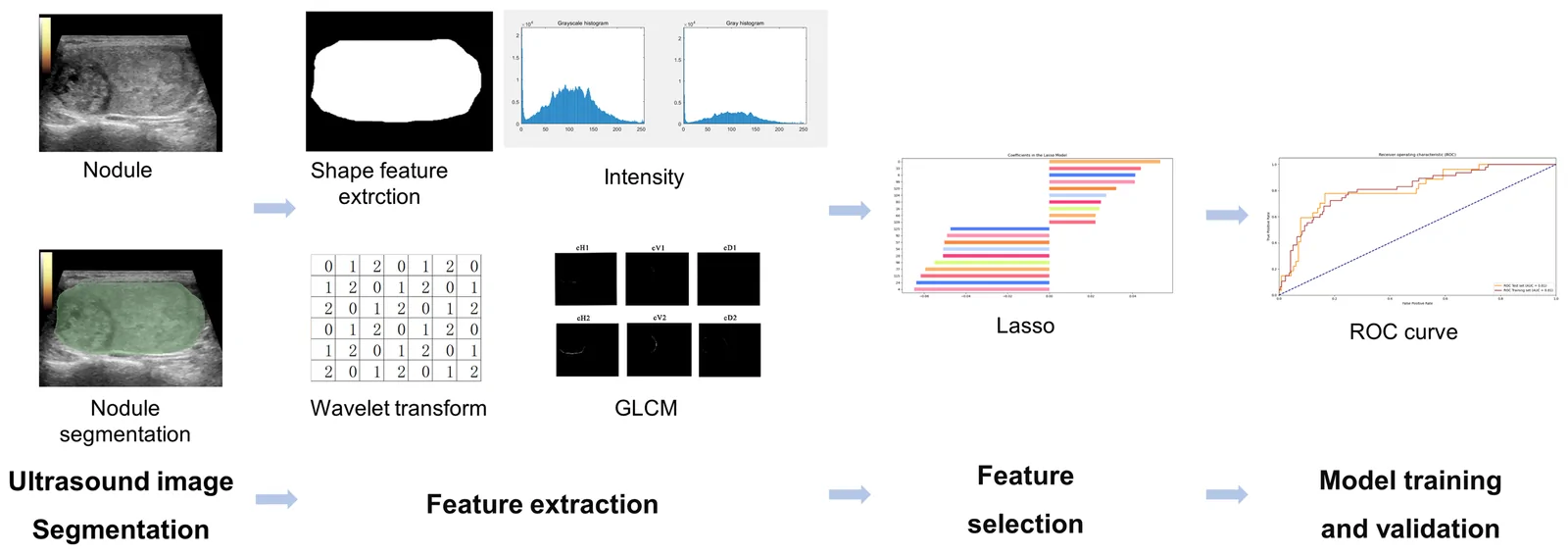
Flowchart of the machine learning-based ultrasound radiomics analysis process. The process involved nodule segmentation, radiomics feature exaction, radiomics feature selection, and model training and validation. cH1, horizontal high-frequency coefficient of scale 1; cV1, vertical high-frequency coefficient of scale 1; cD1, diagonal high-frequency coefficient of scale 1; cH2, horizontal high-frequency coefficient of scale 2; cV2, vertical high-frequency coefficient of scale 2; cD2, diagonal high-frequency coefficient of scale 2; GLCM, gray-level co-occurrence matrix; LASSO: least absolute shrinkage selection operator; ROC, receiver operating characteristic.

Based on the best image features obtained, five ML algorithms—support vector machine (SVM), logistic regression, decision tree, K-nearest neighbors (KNN), and random forest—were used for image classification. In the training cohort, the model was trained using 20-*fold* cross-validation. The cross-validation first divided the data set into a 70% training cohort and 30% test cohort before 20-*fold* cross-validation on training cohort data. First, the initial sample was split into K subsamples; a single subsample was kept as validation data and the other K–1 samples were used for training. The cross-validation was then repeated K times (once per subsample), and the results averaged over K times or combined using other methods to obtain a single estimate. The advantage of this method is that it repeatedly uses randomly generated subsamples for training and verification and the results are verified each time. Then, the features were fed into the model, and a radiomics model was built on training data for VRR< 50% and VRR ≥ 50% at 6-month after TA. A receiver operating characteristic (ROC) curve was used to analyze and evaluate model performance. Diagnostic efficacy was quantified using the area under the curve (AUC). A model training and testing flowchart is shown in **Figure 1**.

### 2.6 Establishment of a clinical and combined model

The influencing factors that may affect the VRR at 6 months after TA were included in a binary logistic regression analysis and the clinical prediction model constructed according to the influencing factors obtained from the multivariate analysis. Both clinical factors and selected US radiomics features were then combined with ML algorithms to construct a combined model. Then, a ROC curve was used to analyze and evaluate model prediction performance. A calibration curve was drawn to measure the model’s reliability and a decision curve to evaluate the clinical applicability of the model based on the determined net benefit.

### 2.7 Statistical methods

Continuous variable data are expressed as mean ± SD or median with 25%–75% interquartile range (IQR), and categorical variable data as numbers (%). The former data was compared using the t-test, Wilcoxon signed-rank test, or Mann–Whitney U test, and the latter using the chi-square test. ROC curve analysis was used to assess the model predictive performance for the mid-term LTR of TA. Main performance indicators included the AUC, accuracy, sensitivity, specificity, positive predictive value (PPV), and negative predictive value (NPV). The AUC was compared between models using the DeLong test. Model sensitivity and specificity were compared using the chi-square test. Statistical analysis was performed using SPSS (version 22.0; SPSS, Chicago, IL, USA) and R software (version 4.0.5; R Foundation for Statistical Computing, Vienna, Austria). Model building and validation were performed using the Python scikit-learn package (ver. 0.24.2). Factors with *P* < 0.2 in univariate analysis were included in multivariate analysis. All other statistical tests were two-sided and the statistical significance level set at *P* < 0.05.

All analyses in this study were performed at the nodule level rather than the patient level. Each thyroid nodule was regarded as an independent research subject, and one ultrasound image at the maximum transverse section of each nodule was selected for ROI segmentation and radiomic feature extraction.

## 3 Results

### 3.1 Basic clinical information

**Figure 2:**
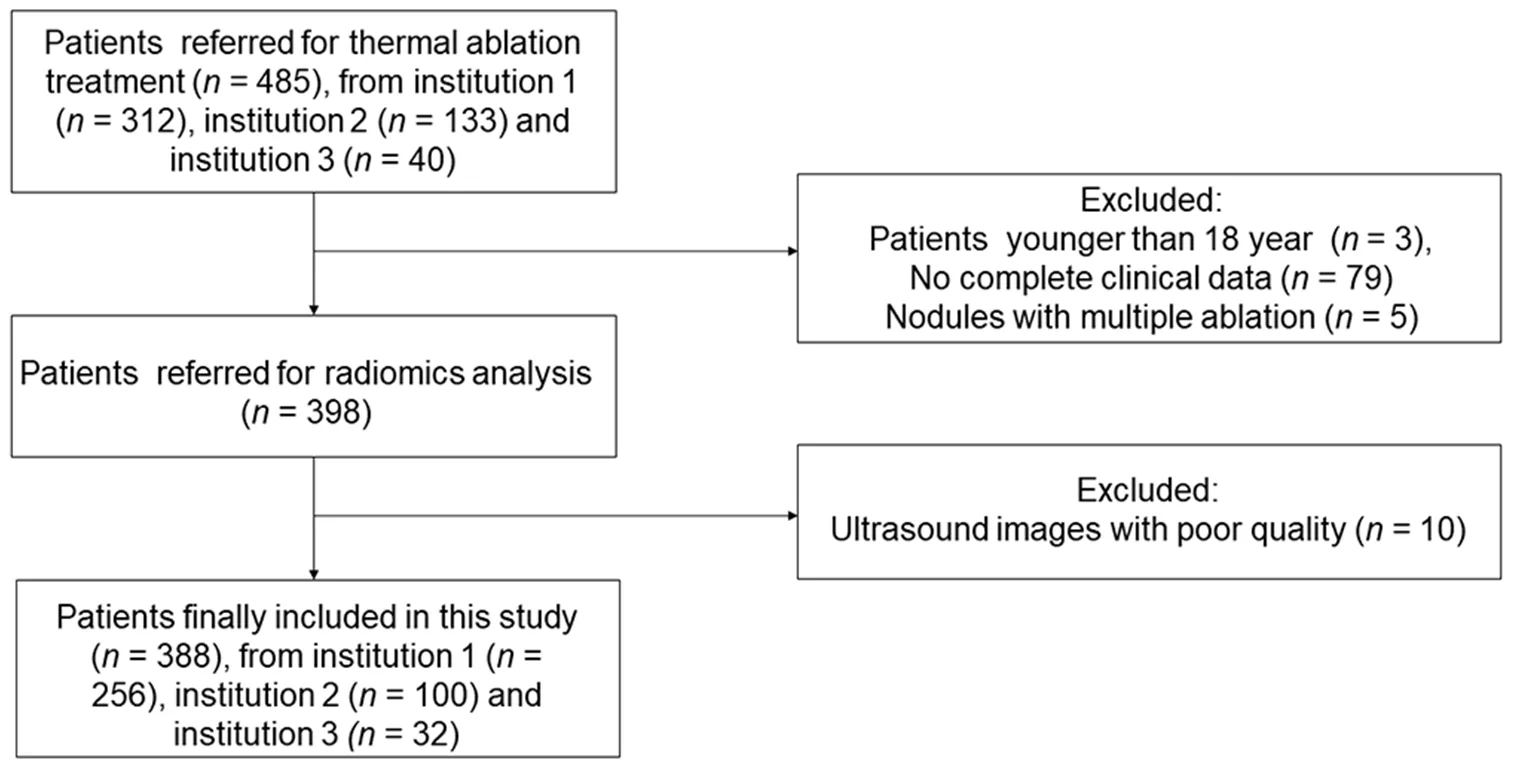
Patient enrollment flowchart.

**Figure 2** shows the enrollment flowchart. In total, 388 patients with 404 nodules were included in this study, of which 356 patients in institutions 1 and 2 had a total of 372 nodules containing 405 US images for model training (283 images) and internal testing (122 images). Among them, Unit 1 had 256 patients with 270 nodules (14 patients had 2 lesions), and Unit 2 included 100 patients with 102 nodules (1 patient had 3 lesions). Institution 3 included a total of 32 patients with 32 nodules for the external test cohort. **Table 1** summarizes the basic clinical characteristics of all patients.

**Table 1:** Baseline clinical and ultrasound feature characteristics of all patients.

Parameter	Training and internal cohort (n = 356)	External test cohort (n = 32)	*P*
Gender, n (%)			0.176
Female	300 (84.3)	24 (75.0)	
Male	56 (15.7)	8 (25.0)	
Age (years), mean ± SD (range)	48.6 ± 13.0 (19–78)	48.6 ± 16.6 (19–82)	0.985
Ablation method, n (%)			<0.001
Radiofrequency ablation	54 (14.5)	32 (100)	
Microwave ablation	318 (85.5)	0 (0)	
Nodule maximum diameter (cm), mean ± SD (range)	3.1 ± 1.2 (1.2–8.3)	3.4 ± 1.0 (1.4–5.2)	0.204
Nodule volume (mL), median (25%–75% IQR)	5.9 (2.3–12.7)	8.1 (4.4–15.3)	0.097
Nodule location, *n* (%)			0.053
Left	184 (49.5)	22 (68.8)	
Right	175 (47.0)	8 (25.0)	
Isthmus	13 (3.5)	2 (6.3)	
Nodules close to vital organs, n (%)	167 (44.9%)	21 (65.6)	0.120
Echo, n (%)			0.025
Hyperechoic	3 (0.8)	0 (0)	
Isoechoic	82 (22.0)	1 (3.1)	
Hypoechoic	38 (10.2)	2 (6.3)	
Mixed	249 (66.9)	29 (90.6)	
Echostructure, n (%)			0.212
Cystic	161 (43.3)	19 (59.4)	
Microcystic	118 (31.7)	7 (21.9)	
Solidity	93 (25.0)	6 (18.8)	
Vascular pattern, n (%)			0.002
Peri- and intranodular intense vascularity	51 (13.7)	5 (15.6)	
Peripheral intense vascularity	30 (8.1)	3 (9.4)	
Peripheral weak vascularity	271 (72.9)	16 (50.0)	
No vascularity	20 (5.4)	8 (25.0)	
Macrocalcifications, n (%)			0.441
Yes	6 (1.6)	1(3.1)	
No	366 (98.4)	31(96.9)	

SD, standard deviation; IQR, inter quartile range.

### 3.2 Patient ablation results

Six months after ablation, the median patient nodule volume decreased from 5.9 mL (IQR, 2.3–12.7 mL) to 1.3 (IQR, 0.4–3.5 mL; *P<* 0.001), and the median patient VRR was 74.5% (IQR, 55.0%–89.0%). The sufficient ablation rate was 75.8% (292/372), and 6 (1.6%, 6/372) nodules completely disappeared in the training and internal test cohorts. The median patient nodule volume in the external test cohort decreased from 8.1 mL (IQR, 4.4–15.3 mL) preoperatively to 2.9 mL (IQR, 1.1–6.6 mL; *P* < 0.001), and the median patient VRR was 53.5% (IQR, 14.5%–88.7%). Nodules with VRR ≥ 50% accounted for 59.4% (19/32), of which 1 (3.1%, 1/32) completely disappeared.

### 3.3 Univariate and multivariate analyses

In this study, 11 factors were analyzed using univariate analysis; those that achieved statistical significance were included in the multivariable analysis (**Table 2**). The results of this second analysis showed that solid nodules (odds radio, 2.52; 95% confidence interval, 1.356–4.683; *P<* 0.01) were the only independent predictors associated with VRR< 50%.

**Table 2:** Univariate and multivariate analyses for risk factors associated with VRR< 50% of thermal ablation for benign thyroid nodules.

Factors	Univariate analysis		Multivariate analysis	
	OR (95% CI)	*P*	OR (95% CI)	*P*
Female	2.26 (0.984–5.189)	0.055	1.956 (0.823–4.648)	0.129
Age	0.986 (0.968–1.006)	0.166	0.992 (0.972–1.012)	0.431
Ablation method (microwave ablation)	Reference			
Ablation method (radiofrequency ablation)	0.565 (0.255–1.249)	0.158	0.569 (0.253–1.279)	0.172
Max diameter of the nodule	0.987 (0.967–1.008)	0.220	–	
Nodule volume	1 (1.000–1.000)	0.112	1 (1.000–1.000)	0.295
Location (left/right)	Reference			
Location (isthmus)	1.099 (0.295–4.091)	0.888	–	
Nodules close to vital organs	0.828 (0.502–1.366)	0.460	–	
Echogenicity (hyper-)	Reference			
Echogenicity (iso-)	0.877 (0.076–10.166)	0.916	–	
Echogenicity (hypo-)	1.04 (0.086–12.573)	0.975	–	
Echogenicity (mixed)	0.394 (0.035–4.45)	0.452	–	
Nodule internal structure (cystic)	Reference			
Nodule internal structure (microcystic)	1.389 (0.748–2.579)	0.298	1.291 (0.683–2.439)	0.431
Nodule internal structure (solidity)	2.72 (1.483–4.988)	0.001	2.52 (1.356–4.683)	0.003
Vascular pattern (none)	Reference			
Vascular pattern (peri- and intense intranodular)	1.214 (0.293–5.039)	0.789	–	
Vascular pattern (intense perinodular)	1.725 (0.388–7.658)	0.474	–	
Vascular pattern (peripheral weak vascularity)	1.646 (0.467–5.803)	0.438	–	
Calcification (none)	Reference		–	
Calcification (macrocalcification)	1.846 (0.332–10.266)	0.484	–	

VRR, volume reduction ratio; OR, odds ratio; CI, confidence interval.

### 3.4 Radiomic feature extraction and ultrasound image selection

A total of 1,409 radiomics features per image were obtained using the PyRadiomics form, including 16 shape and imaging features extracted from the original and derived images. Among these, 72.0% (1015/1409) of the features had ICC ≥ 0.8. Based on PCA, a total of 150 radiomics features were selected. Based on the Lasso regression model, the optimal λ in cross-validation was used to select the best radiomic features with non-zero coefficients, and the 10 best features were selected (**Figure 1**).

### 3.5 Performance of the three models in training and internal test cohorts

**Figure 3:**
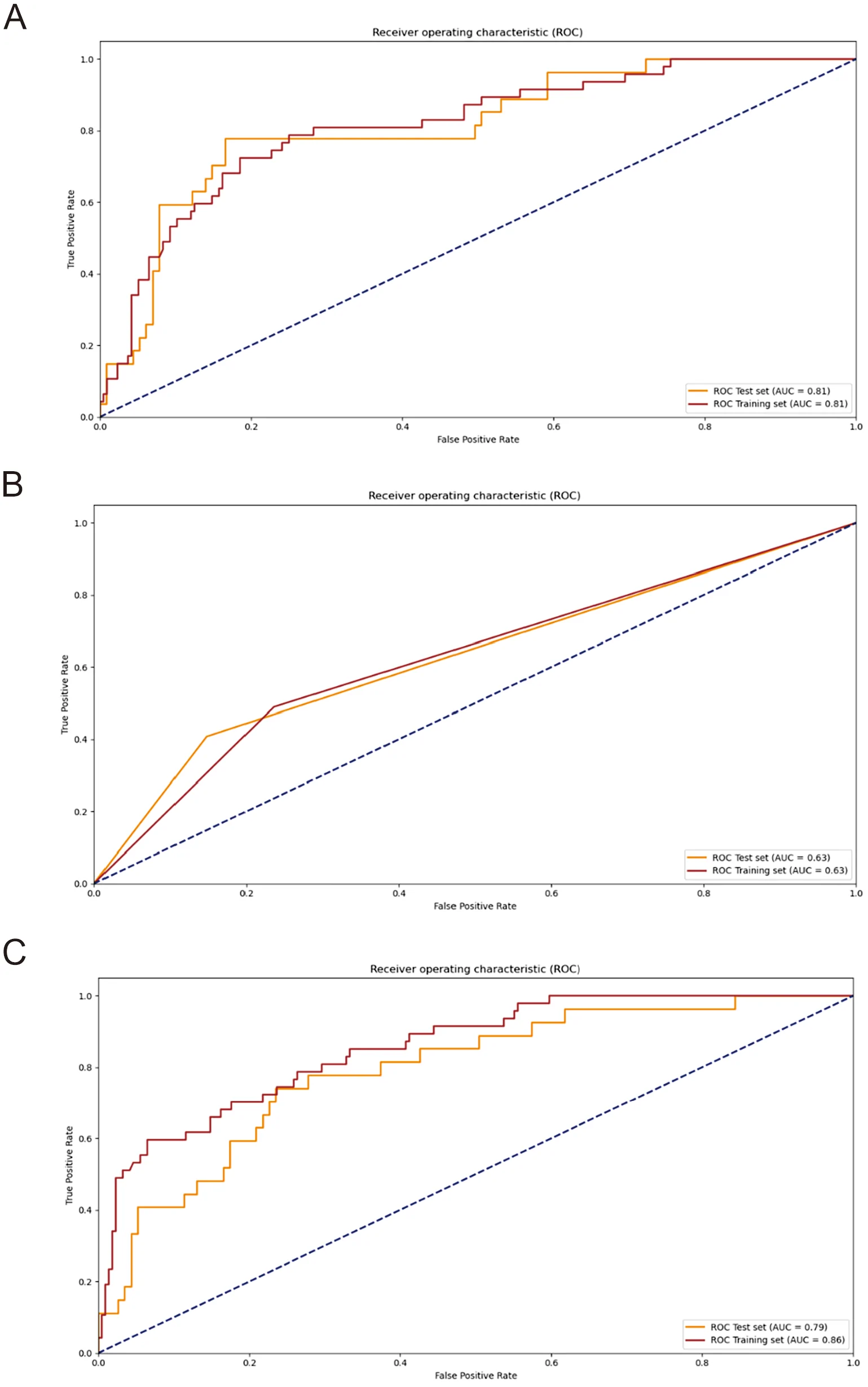
ROC curves of different models for differentiating benign thyroid nodules VRR ≥ 50% or < 50% after ablation. **(A)** ROC curves of the SVM algorithm in training and internal test cohorts. **(B)** ROC curves of the clinical model in training and internal test cohorts. **(C)** ROC curves of the combined model in training and internal test cohorts. (ROC, receiver operating characteristic; SVM, support vector machine).

Among the five ML algorithms, the highest AUC in the internal test cohort was that of the SVM; the AUC, accuracy, sensitivity, specificity, PPV, and NPV were 0.81, 0.82, 0.78, 0.83, 0.53, and 0.94, respectively. Specific performance data are listed in **Table 3**. The KNN was overfitted in the training cohort; therefore, those values were absent. **Figure 3A** shows the ROC curves of the ML- SVM in the training and internal test cohorts.

**Table 3:** Performance of various models for local treatment response prediction of TA for BTNs in both training and internal test cohorts.

Metric	Cohort	SVM	LR	DT	KNN	RF
AUC (95%CI)	Training cohort	0.81 (0.76–0.87)	0.76 (0.70–0.81)	0.65 (0.60–0.71)	*	0.82 (0.77–0.87)
	Internal test cohort	0.81 (0.77–0.86)	0.77 (0.70–0.81)	0.64 (0.56–0.70)	0.68 (0.62–0.73)	0.65 (0.59–0.70)
Accuracy (95% CI)	Training cohort	0.76 (0.74–0.80)	0.68 (0.65–0.72)	0.63 (0.60–0.67)	*	0.87 (0.85–0.89)
	Internal test cohort	0.82 (0.80–0.85)	0.76 (0.74–0.80)	0.62 (0.58–0.66)	0.76 (0.74–0.79)	0.78 (0.75–0.81)
Sensitivity (95% CI)	Training cohort	0.74 (0.67–0.83)	0.73 (0.66–0.84)	0.82 (0.76–0.90)	*	0.49 (0.41–0.59)
	Internal test cohort	0.78 (0.72–0.86)	0.70 (0.60–0.78)	0.70 (0.60–0.79)	0.39 (0.32–0.49)	0.21 (0.15–0.31)
Specificity (95% CI)	Training cohort	0.77 (0.66–0.83)	0.67 (0.63–0.82)	0.59 (0.76–0.90)	*	0.50 (0.40–0.60)
	Internal test cohort	0.83 (0.79–0.87)	0.78 (0.74–0.81)	0.61 (0.56–0.64)	0.85 (0.81–0.88)	0.91 (0.88–0.93)
PPV (95% CI)	Training cohort	0.41 (0.37–0.47)	0.33 (0.29–0.36)	0.30 (0.28–0.35)	*	0.69 (0.62–0.79)
	Internal test cohort	0.53 (0.48–0.58)	0.42 (0.38–0.47)	0.29 (0.25–0.33)	0.38 (0.30–0.44)	0.36 (0.24–0.45)
NPV (95% CI)	Training cohort	0.93 (0.92–0.95)	0.92 (0.90–0.95)	0.94 (0.91–0.96)	*	0.89 (0.88–0.92)
	Internal test cohort	0.94 (0.93–0.96)	0.92 (0.89–0.97)	0.90 (0.87–0.92)	0.87 (0.84–0.88)	0.83 (0.82–0.84)

TA, thermal ablation; BTNs, benign thyroid nodules; CI, confidence interval; AUC, area under the curve; PPV, positive predictive value; NPV, negative predictive value.

*The KNN model exhibited severe overfitting, with a marked decline in AUC from training to internal validation set.

**Figure 4:**
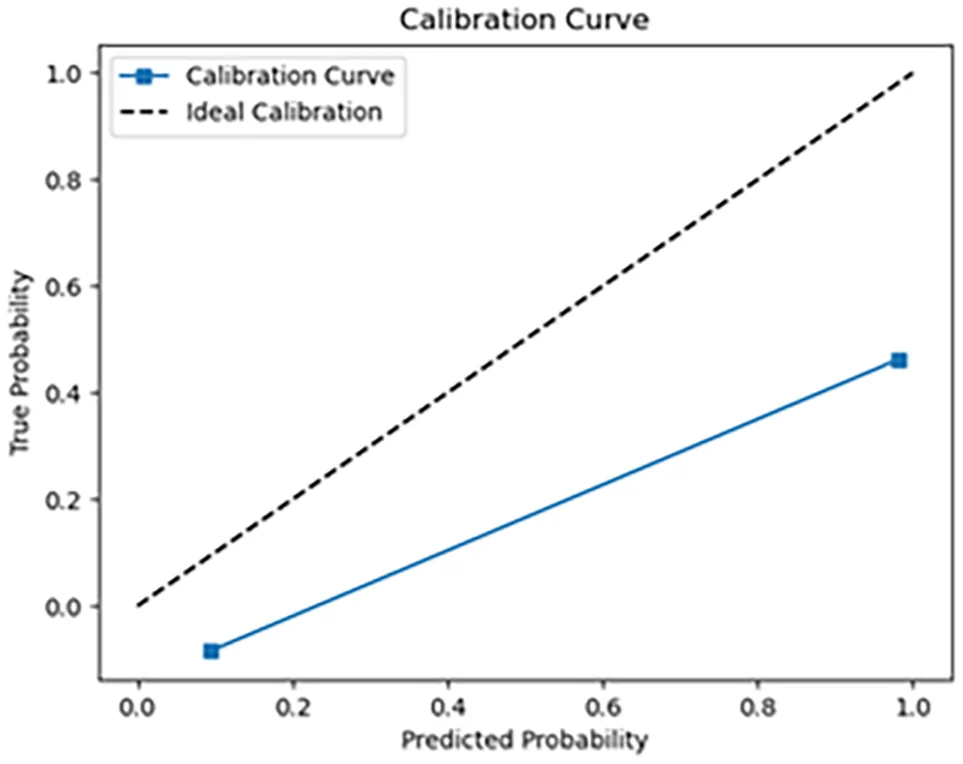
Calibration curve for the combined model.

Ten radiomic features and one clinical factor (i.e., solid nodule) were combined to construct a combined model. **Table 4** shows the AUC, accuracy, sensitivity, specificity, PPV, and NPV of the combined and clinical models in training and test cohorts. In the internal test cohort, the AUC of the combined and ML-SVM models was comparable (0.79 vs. 0.81, *P* = 0.899), but both were significantly higher than in the clinical model alone (0.79 vs. 0.63, *P<* 0.01; 0.81 vs. 0.63, *P<* 0.05). ML-SVM model’s sensitivity was significantly higher than that of the combined and clinical models (0.78 vs. 0.41, *P<* 0.01; 0.78 vs. 0.48, *P<* 0.01), which was comparable (0.41 vs. 0.48, *P* = 0.246). However, the specificity of the ML-SVM (0.83), combined (0.85), and clinical (0.84) models was similar (*P* = 0.868). **Figures 3B, C** show ROC curves for the clinical and combined models. The calibration curve (**Figure 4**) shows good calibration performance for the combined model.

**Table 4:** Performance of various models for local treatment response prediction of TA for BTNs in both training and internal test cohorts.

Model	Cohort	AUC (95%CI)	Accuracy (95%CI)	Sensitivity (95%CI)	Specificity (95%CI)	PPV (95%CI)	NPV (95%CI)
Clinical model	Training cohort	0.63 (0.57–0.67)	0.71 (0.68–0.76)	0.49 (0.38–0.59)	0.49 (0.41–0.62)	0.31 (0.27–0.36)	0.87 (0.86–0.89)
	Internal test cohort	0.63 (0.58–0.69)	0.77 (0.74–0.79)	0.41 (0.34–0.51)	0.85 (0.82–0.89)	0.39 (0.33–0.48)	0.86 (0.84–0.88)
Combined model	Training cohort	0.86 (0.82–0.89)	0.79 (0.76–0.83)	0.70 (0.63–0.79)	0.70 (0.62–0.79)	0.44 (0.38–0.50)	0.92 (0.90–0.95)
	Internal test cohort	0.79 (0.73–0.85)	0.77 (0.75–0.81)	0.48 (0.37–0.57)	0.84 (0.82–0.88)	0.42 (0.37–0.49)	0.87 (0.86–0.89)

TA, thermal ablation; BTNs, benign thyroid nodules; CI, confidence interval; AUC, area under the curve; PPV, positive predictive value; NPV, negative predictive value.

### 3.6 Performance of the three models in external test cohorts

Specific performance data for the three models is shown in **Table 5**. In an independent external test cohort, the AUC of the ML-SVM radiomics model and the combined model were similar (0.77 vs. 0.77, *P* = 0.945); both outperformed the clinical model (0.77 vs. 0.54, *P<* 0.05; 0.77 vs. 0.54, *P* < 0.01). The ML-SVM radiomics and clinical models had similar sensitivity (0.38 vs. 0.28, *P* = 0.021), but higher specificity (0.95 vs. 0.84, *P* < 0.01). The combined model had higher sensitivity than the clinical model (0.46 vs. 0.28, *P* < 0.01), with similar specificity (0.89 vs. 0.84, *P* = 0.300).

**Table 5:** Performance of various models for local treatment response prediction of TA for BTNs in the external test cohort.

Metric	ML-SVM radiomics	Clinical model	Combined model	*P*
AUC (95% CI)	0.77 (0.72–0.81)	0.54 (0.50–0.58)	0.77 (0.73–0.81)	<0.001
Accuracy (95% CI)	0.72 (0.69–0.75)	0.59 (0.57–0.63)	0.72 (0.69–0.75)	/
Sensitivity (95% CI)	0.38 (0.34–0.46)	0.23 (0.18–0.28)	0.46 (0.39–0.52)	0.003
Specificity (95% CI)	0.95 (0.92–0.97)	0.84 (0.80–0.88)	0.89 (0.87–0.92)	0.041
PPV (95% CI)	0.83 (0.77–0.90)	0.50 (0.42–0.59)	0.75 (0.70–0.82)	/
NPV (95% CI)	0.69 (0.67–0.71)	0.62 (0.60–0.63)	0.71 (0.69–0.74)	/

TA, thermal ablation; BTNs, benign thyroid nodules; CI, confidence interval; AUC, area under the curve; PPV, positive predictive value; NPV, negative predictive value.

AUC: ML-SVM radiomics compared with clinical model, *P* = 0.035; ML-SVM radiomics compared with combined model, *P* = 0.945; clinical model compared with combined model, *P* = 0.007.

Sensitivity: ML-SVM radiomics compared with the clinical model, *P* = 0.021; ML-SVM radiomics compared with the combined model, *P* = 0.251; clinical model compared with the combined model, *P* = 0.001.

Specificity: ML-SVM radiomics compared with clinical model, *P* = 0.009; ML-SVM radiomics compared with combined model, *P* = 0.114; clinical model compared with combined model, *P* = 0.30.

### 3.7 Clinical application

**Figure 5:**
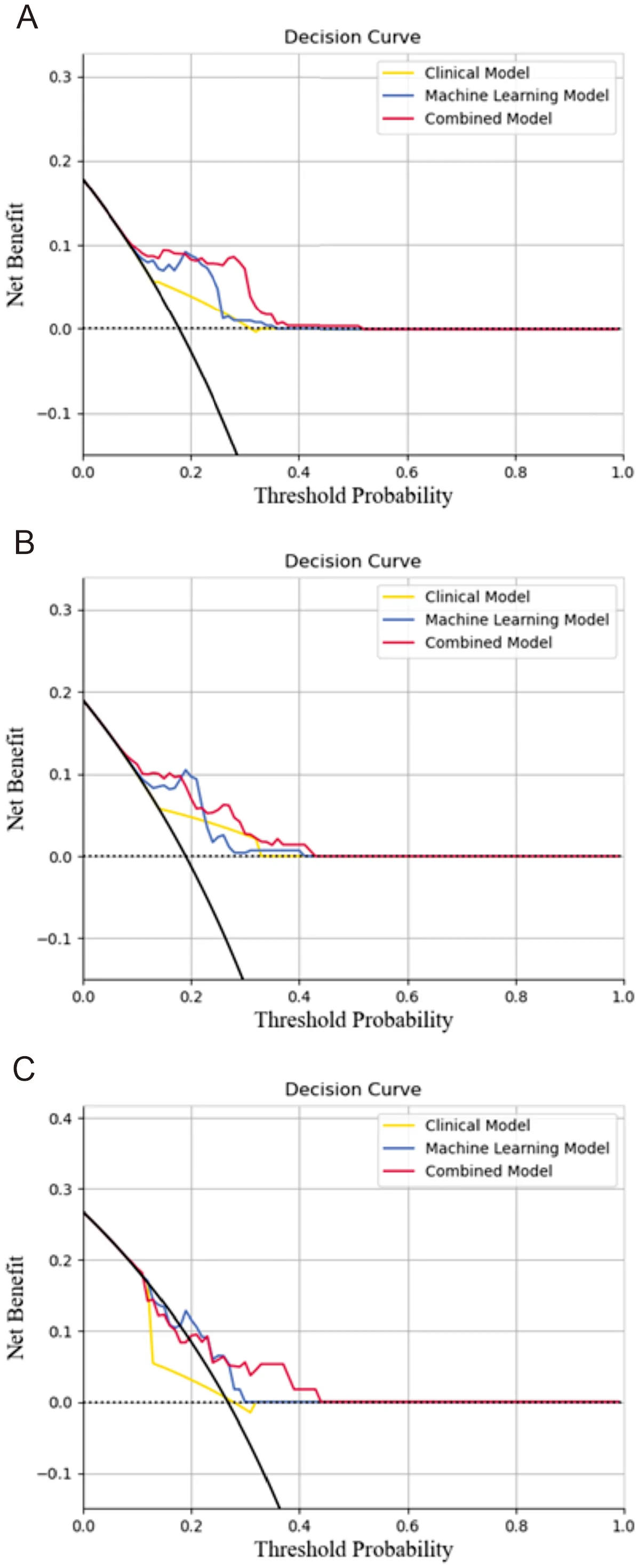
Decision curve analysis of clinical, machine learning, and combined models. **(A)** training cohort; **(B)** internal test cohort; and **(C)** external test cohort. Solid and dashed black lines represent all patients undergoing the “treat all” or “treat none” strategy, respectively. Yellow curves represent the clinical model, blue ones the machine learning model, and red ones the combined model.

The decision curves (**Figure 5**) show that the combined model can result in greater net benefits relative to both ML-SVM radiomics and clinical models in the threshold probability range.

## 4 Discussion

In this study, we used ML-based US radiomics features to predict the LTR of TA in patients with BTNs at 6 months after TA. Our findings indicate that the ML and combined models can precisely predict a VRR ≥ 50% with better performance than the clinical model in the internal and external test cohorts.

The ML model can assist in therapy decision-making. At present, TA has been widely recommended, showing favorable results; however, not all nodules are suitable for ablation, and some shrink slowly or regrow over time. Doctors make general judgments based on their experience. However, subjective judgment is often biased. Solidity was the only factor affecting the LTR, as previously reported (22, 24). However, another study suggested that nodules with predominantly cystic or solid parts have comparable efficacy (25). Several studies have shown that larger nodule volume, macrocalcifications, and output ablation energy are risk factors (3, 22, 24, 26). Accordingly, no reliable or objective risk factors can predict 6-month LTR. In this study, the prediction accuracy of the clinical model based on LTR-influencing factors was lower than that of the combined and ML-based radiomics models. The ML-based radiomics model could allow evaluating whether ablation is effective before TA to choose the most suitable treatment method. Radiomics methods can extract a large amount of information from medical images and rate it by training ML algorithms. In recent years, radiomics and ML methods have been explored in clinical practice and have shown good clinical value (27–31).

Colakoglu et al. (27) showed that ML-based quantitative texture analysis could accurately identify 87 of 102 malignant TNs and 117 of 133 BTNs, with a sensitivity of 85.2%, specificity of 87.9%, accuracy of 86.8%, and AUC of 0.92. Some studies have suggested that US-based or ML-based US radiomics can help distinguish benign and malignant parotid gland lesions, breast lesions, and primary and metastatic liver cancer (28–30). Similarly, our study showed that the ML-based US radiomics model has potential for clinical application in the prediction of postoperative efficacy after TA.

In a study on ML in TA for patients with BTNs, Negro et al. (32) developed an automatic ML model based on nodular volume, echostructure, macrocalcifications, vascularity, and 12-month VRR, which can help predict efficacy at one year after RFA with 85% accuracy. However, these variables were independently established and could not objectively reflect the internal high-flux information of nodules. In comparison, our study was based on the extracting imaging radiomic features from US images, and an ML model was established to identify VRR ≥ 50% after TA, with an accuracy of 82% and 72% in the internal and external test cohorts. Despite the slightly lower accuracy of our model, it was more objective. Moreover, our study could predict the LTR of TA at an early stage. Another study (33) developed an US-based radiomics model to predict the VRR of BTNs at VRR<75% or ≥75% at 12 months after MWA, with AUC values in the validation cohort of 0.820, 0.844, and 0.917, respectively. In comparison, our study includes a larger sample size and an external test cohort.

Nonetheless, this study had some limitations. First, it was a retrospective study and selection bias inevitable. Second, RFA and MWA were used. Although some studies have shown similar ablation efficacies for the two techniques (34, 35), more data are required to separately test model performance. Further, there was an imbalance of ablation modalities between the training and external validation cohorts. Due to the limited sample size of the external cohort, stratified subgroup analysis could not be performed. However, the radiomic features were derived from preoperative images and would not be affected by the postoperative ablation approaches. In addition, RFA and MWA present similar 6-month outcomes for BTNs of small and medium size. Further studies with balanced treatment modalities are required to validate our findings. Third, only grayscale US images were used for radiomics feature extraction; multimodal ultrasound, such as CDFI and elastography, may provide more information. Fourth, this study is based on LTR prediction at 6 months after TA and not ≥1 year. According to previous studies, the most obvious volume shrinkage of BTNs occurs within one year after ablation, and 6-month efficacy can reflect early ablation response. However, nodule regrowth generally appears 2–3 years after treatment. Limited by retrospective data, we could not perform correlation analysis between 6- and 12-month outcomes. Our radiomics model is suitable for early auxiliary evaluation of treatment response and cannot be used to predict long-term nodule regrowth. Future studies with complete long-term follow-up data are needed for further validation. Lastly, the adoption of single-slice segmentation cannot completely reflect the 3D heterogeneity of thyroid nodules; multi-slice or 3D segmentation is superior in capturing whole-lesion information. Future work will apply volumetric ROI delineation to improve model performance.

Treatment response was dichotomized by a 50% VRR cutoff. Although this threshold is guideline-endorsed and widely applied, disparate cutoffs exist in published works. Symptom improvement and cosmetic outcomes were recorded but lacked unified quantitative grading across centers, precluding their incorporation into the predictive model. US examinations were performed on two different US machines without fully standardized acquisition settings. The available ICC values only represent interobserver reproducibility within a single center; cross-device feature reproducibility and ComBat harmonization analysis could not be performed due to incomplete original archived DICOM files. A standardized imaging protocol will be used in future prospective research. One limitation is the lack of internal validation of the 50% VRR cutoff using long-term follow-up data. This threshold was referenced from authoritative guidelines and previous clinical evidence rather than verified by the 12-month outcomes of our cohort, as complete 12-month imaging was not available for all cases. Further prospective trials with consistent long-term follow-up are needed to validate this cutoff value.

## 5 Conclusion

The proposed ML-based US radiomics model had good performance in predicting the efficacy of TA, better than the clinical model. This model provides a new solution for preoperative prediction of TA efficacy in patients with BTNs, thereby facilitating decision-making.

## Data Availability

The raw data supporting the conclusions of this article will be made available by the authors, without undue reservation.
